# Editorial: Interneurons in pathological conditions

**DOI:** 10.3389/fncel.2026.1807738

**Published:** 2026-02-27

**Authors:** Helene Lacaille, Claire-Marie Vacher

**Affiliations:** Department of Pediatrics, Columbia University Medical Center, New York, NY, United States

**Keywords:** basolateral amygdala, cerebral cortex, GABA, hippocampus, interneuron, organoid, schizophrenia, sex differences

Interneurons are heterogeneous GABAergic cells that regulate neuronal networks through inhibitory neurotransmission. By maintaining the balance between excitation and inhibition and regulating synchrony and oscillatory rhythms, they coordinate complex cognitive processes including working memory, sensory processing, and emotional regulation. When interneuron function is compromised—whether through genetic predisposition, developmental abnormalities, or acquired injury—the consequences manifest as diverse neurological and psychiatric conditions such as epilepsy, schizophrenia, autism spectrum disorders, affective disorders, Down syndrome, Alzheimer's disease, and traumatic brain injury.

**Figure 1:**
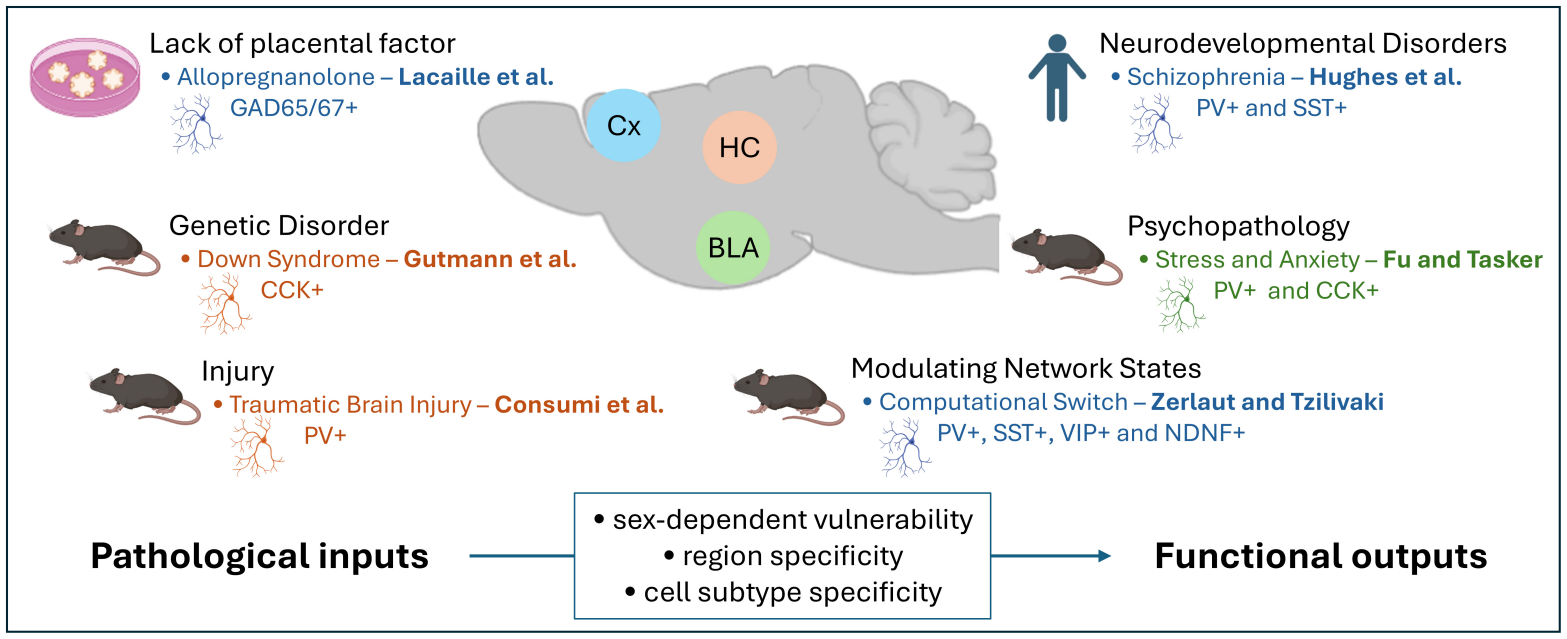
The multi-dimensional landscape of interneuron pathophysiology. This research topic integrates findings from six studies across organoid, mouse, and human post-mortem models to demonstrate that interneuron dysfunction is highly specific to sex, subtype, and region (Cx, cerebral cortex, blue; HC, hippocampus, orange; BLA, basolateral amygdala, green). The framework illustrates how primary drivers, including developmental abnormalities, genetic predispositions, and acquired injuries, converge to produce pathological outcomes across a spectrum of neurological and psychiatric conditions. Crucially, these contributions reveal that pathology stems from disrupted network dynamics, impaired dendritic computation, and the failure of neuromodulatory systems to adaptively reconfigure circuits, rather than simple cell loss.

This Research Topic brings together six articles examining how interneuron dysfunction contributes to pathological conditions while revealing novel mechanisms through which these cells enable adaptive network computations (Figure 1). Understanding interneuron pathophysiology requires integrating insights across multiple scales—from molecular alterations to circuit dynamics and behavioral outcomes—with particular attention to subtype-specific vulnerabilities and sex-dependent mechanisms.

Down syndrome is the most common genetic cause of intellectual disability. Individuals face elevated risks for epilepsy and early-onset Alzheimer's, yet effective treatments for cognitive impairments remain unavailable. The Ts65Dn mouse, featuring a segmental trisomy of distal chromosome 16, serves as the primary experimental system for studying underlying mechanisms. Excitation–inhibition imbalance is considered as a key contributor to the learning and memory deficits in Down syndrome. Ts65Dn mice show heightened dentate gyrus miniature IPSC frequency and enhanced GABA-A and GABA-B receptor-mediated IPSCs, but which interneuron subtypes drive this excess inhibition was unknown. Using optogenetic, electrophysiological, and histological approaches, Gutmann et al. revealed a complex inhibitory dysregulation in the Ts65Dn dentate gyrus. Their study suggests that excitation–inhibition imbalance in Down syndrome involves redistribution of inhibitory control along the dendritic axis rather than a global shift and highlights differential effects across interneuron subtypes in neurodevelopmental disorders.

A notable theme emerging from this Research Topic is the sex-specific vulnerability of interneurons to pathological insults. Consumi et al. demonstrate that traumatic brain injury produces markedly different effects in the hippocampus of male vs. female rodents. Females show greater interneuron loss and elevated pro-apoptotic signaling yet display more transient cognitive deficits than males. Males exhibit sustained cognitive impairments associated with reduced pro-survival mechanisms. This dissociation between cellular pathology and functional outcomes challenges simplistic models of interneuron dysfunction and underscores an urgent need for sex-informed therapeutic strategies that recognize distinct mechanistic pathways converging on similar behavioral phenotypes.

In schizophrenia, interneuron dysfunction shows subtype-specific patterns that impair working memory. Hughes et al. review the molecular and cellular alterations in interneuron pathology in schizophrenia, focusing on the dorsolateral prefrontal cortex, a region critical for working memory. Their analysis reveals a nuanced picture of subtype-specific alterations: some interneuron subtypes show reduced markers suggesting decreased functionality, while others are relatively spared. These changes occur alongside broader GABAergic dysfunction that collectively impairs neural synchronization underlying working memory. Importantly, Hughes et al. note that affective disorders including bipolar disorder and major depressive disorder show inconsistent interneuron pathology, with region- and layer-specific effects that differ from schizophrenia, suggesting that while these conditions share cognitive symptoms, their underlying circuit-level mechanisms may diverge. Elucidating the precise nature of GABAergic abnormalities (including direct measurement of GABA levels in human brain tissue) and their consequences for neural circuit dysfunction in schizophrenia remains essential for designing therapeutics that target specific interneuron subtypes and restore functional network dynamics.

Fu and Tasker add another dimension by examining how neuromodulatory systems regulate interneuron function in the basolateral amygdala (BLA) during emotional states. In this region, stress-induced anxiety involves interactions between glucocorticoid and endocannabinoid signaling at inhibitory synapses. This review describes how multiple neuromodulators converging on Gq-coupled signaling pathways produce cell type-specific activity patterns. These activation patterns, generalizable across different neurotransmitters and receptors, represent a fundamental mechanism for tuning BLA network oscillations and fear expression. The findings challenge the common assumption that Gq-coupled Designer Receptors Exclusively Activated by Designer Drugs (DREADDs) simply increase neural excitability. Instead, neuromodulation introduces specific patterns of network activation that may reflect different operational modes of circuit function.

Zerlaut and Tzilivaki offer a fresh perspective on cortical inhibition, proposing that beyond their classical roles in network stability and temporal precision, certain interneuron subtypes act as computational switches, dynamically reconfiguring how cortical circuits process information. Drawing on experimental and theoretical evidence from rodent sensory cortices, they argue that interneuron dysfunction may impair this computational flexibility, contributing to neurodevelopmental and psychiatric disorders. They highlight emerging evidence that specific interneuron subtypes possess sophisticated dendritic integration and plasticity properties. Understanding these cell-type-specific mechanisms will be essential for developing targeted interventions that restore adaptive circuit function in disease.

Using human cortical organoid technology, Lacaille et al. demonstrate that withdrawal of the placental neurosteroid allopregnanolone selectively disrupts GABAergic interneuron development while leaving glutamatergic neurons largely unaffected. This cell type-specific vulnerability provides a mechanistic explanation for the increased neurodevelopmental risk observed in preterm infants, who experience abrupt cessation of placental allopregnanolone support weeks before term. The organoid platform not only allows to study disease mechanisms but also provides an experimental system for testing candidate interventions and dissecting their cellular and molecular mechanisms in human tissue.

These contributions advance understanding of interneuron pathophysiology in three critical ways. First, interneuron dysfunction involves subtype-specific, region-specific, and sex-specific alterations. Second, functional consequences depend on how alterations affect network dynamics and computational properties, not just cell loss or molecular changes. Third, neuromodulatory systems dynamically regulate interneuron function, suggesting pathology may reflect impaired adaptive network reconfiguration.

Future research priorities include thorough characterization of interneuron plasticity mechanisms and dendritic computation capabilities. Understanding how molecular alterations translate to circuit dysfunction requires integrating post-mortem human studies, dynamic animal model recordings, and *ex vivo* systems such as brain organoids. Targeted therapeutic strategies must account for sex differences, subtype specificity, and computational roles.

This Research Topic demonstrates that interneurons are sophisticated computational units whose dysfunction contributes to diverse neurological and psychiatric conditions. The recognition of sex-specific vulnerabilities demands that future research systematically incorporate sex as a biological variable from molecular mechanisms to therapeutic development.

